# Health care providers’ knowledge of clinical protocols for postpartum hemorrhage care in Kenya: a cross-sectional study

**DOI:** 10.1186/s12884-022-05128-6

**Published:** 2022-11-10

**Authors:** Junita Henry, Emma Clarke-Deelder, Dan Han, Nora Miller, Kennedy Opondo, Monica Oguttu, Thomas Burke, Jessica L. Cohen, Margaret McConnell

**Affiliations:** 1 Institute for Life Course Health Research, Department of Global Health, Stellenbosch University, Stellenbosch, South Africa; 2 Department of Global Health and Population, Harvard T. H. Chan School of Public Health , Boston, MA USA; 3 Department of Epidemiology and Public Health, Swiss Tropical and Public Health Institute, Allschwil, Switzerland; 4 Lee Kuan Yew School of Public Policy, National University of Singapore, Singapore, Singapore; 5 Kisumu Medical and Education Trust, Kisumu, Kenya; 6 Global Health Innovation Laboratory, Department of Emergency Medicine, Massachusetts General Hospital, Boston, MA USA; 7 Harvard Medical School, Boston, MA USA

**Keywords:** Postpartum hemorrhage, Health care providers, Knowledge, Sub-Saharan Africa

## Abstract

**Background:**

Postpartum hemorrhage (PPH) remains the leading cause of maternal death worldwide despite its often-preventable nature. Understanding health care providers’ knowledge of clinical protocols is imperative for improving quality of care and reducing mortality. This is especially pertinent in referral and teaching hospitals that train nursing and medical students and interns in addition to managing emergency and referral cases.

**Methods:**

This study aimed to (1) measure health care providers’ knowledge of clinical protocols for risk assessment, prevention, and management of PPH in 3 referral hospitals in Kenya and (2) examine factors associated with providers’ knowledge. We developed a knowledge assessment tool based on past studies and clinical guidelines from the World Health Organization and the Kenyan Ministry of Health. We conducted in-person surveys with health care providers in three high-volume maternity facilities in Nairobi and western Kenya from October 2018-February 2019. We measured gaps in knowledge using a summative index and examined factors associated with knowledge (such as age, gender, qualification, experience, in-service training attendance, and a self-reported measure of peer-closeness) using linear regression.

**Results:**

We interviewed 172 providers including consultants, medical officers, clinical officers, nurse-midwives, and students. Overall, knowledge was lowest for prevention-related protocols (an average of 0.71 out of 1.00; 95% CI 0.69–0.73) and highest for assessment-related protocols (0.81; 95% CI 0.79–0.83). Average knowledge scores did not differ significantly between qualified providers and students. Finally, we found that being a qualified nurse, having a specialization, being female, having a bachelor's degree and self-reported closer relationships with colleagues were statistically significantly associated with higher knowledge scores.

**Conclusion:**

We found gaps in knowledge of PPH care clinical protocols in Kenya. There is a clear need for innovations in clinical training to ensure that providers in teaching referral hospitals are prepared to prevent, assess, and manage PPH. It is possible that training interventions focused on learning by doing and teamwork may be beneficial.

**Supplementary Information:**

The online version contains supplementary material available at 10.1186/s12884-022-05128-6.

## Background

Although maternal mortality has declined by nearly 50% over the past thirty years, it remains high in low- and middle-income countries [1, 2]. Two-thirds of maternal deaths occur in Sub-Saharan Africa (SSA) [2–4].

PPH is the leading cause of maternal death despite its often-preventable nature and the availability of effective treatments for cases when it does occur [5–9]. In Kenya, one in five maternal deaths are attributable to postpartum hemorrhage (PPH) [10]. There is growing evidence that low quality of care, such as low adherence to evidence-based protocols for preventive and curative care, delays in referrals, and delays in provider decision-making during emergencies, is an important contributor to maternal mortality [11–15]. To reduce mortality from PPH, early risk factor identification, implementation of evidence-based preventative care interventions, and timely and correct management are essential [5, 15–17].

A knowledgeable health workforce is an essential ingredient in the provision of high quality care. The existing literature on knowledge of PPH protocols in SSA has focused mainly on preventive care through active management of the third stage of labor (AMSTL), with little in-depth study of knowledge of PPH management. Additionally, past studies have generally used brief self-administered provider questionnaires [8, 9, 18–20]. More evidence is needed on gaps in health providers' knowledge of comprehensive clinical protocols for PPH risk assessment, prevention, and management. In particular, understanding knowledge gaps among providers in teaching and referral hospitals is important due to their roles in training students and managing obstetric emergencies referred from the community and primary health centers. While a growing literature on “know-do gaps” has shown that knowledge alone is not sufficient for high quality care [14, 21, 22], providers’ knowledge levels generally create an upper bound on the quality of care they can provide.

In this paper, we examined provider knowledge of PPH care protocols in three high-volume, regional referral and training hospitals in Kenya. We aimed to (1) describe knowledge of clinical protocols, identifying gaps in knowledge of PPH risk assessment, prevention, and management protocols, and (2) examine factors associated with knowledge in these different domains.

## Methods

### Study setting and design

From 2009 to 2019, Kenya’s maternal mortality rate (MMR) decreased slightly from 309 deaths per 100,000 live births to 280 deaths per 100,000 live births [23]. This decline may be partly attributed to a series of policy changes that made maternity care services more accessible in Kenya during this period. Starting in 2013, the Kenyan government introduced free maternity services in all public facilities, leading to an increase in the rate of deliveries in public health facilities and in the use of postnatal care in public health facilities [24]. Additionally in 2013, health services were devolved from the national government to the county government, which may have contributed to changes such as increased construction of health facilities (particularly levels 2 and 3), increases in the number of specialists, and increased accessibility of skilled delivery services. In 2017, the Kenyan Ministry of Health then launched the “*Linda Mama*” programme, which was designed to further increase access to delivery services. While several challenges have been reported with these programs – including a lack of support for the costs of referrals, challenges for patients trying to access the services to which they were entitled [25], and persistent socioeconomic disparities in access to care [26]– these changes have been associated with improvements in the continuity of care [26]. However, alongside these improvements, several studies have documented important gaps in the quality of maternity care in Kenya [27–29].

We conducted a cross-sectional study among health care providers in three high-volume referral facilities in Nairobi and western Kenya. Data were collected as part of a larger study on postpartum hemorrhage from October 2018 to February 2019. The research was approved by the Harvard University Institutional Review Board (#IRB00047360) and the Ethics and Research Committee of the Jaramogi Oginga Odinga Teaching and Referral Hospital in Kisumu, Kenya. All providers gave their written informed consent for participation.

Kenya’s healthcare system is divided into six levels: 1) Community Health Unit (mostly managed by Community Health Volunteers and Community Health Workers), 2–3) Primary health care facilities (Dispensaries and Health Centres), 4) primary referral facilities/hospitals, 5) secondary referral facilities/hospitals and 6) tertiary referral facilities. The three study facilities serve as level 5 facilities and are both regional referral and training hospitals. These hospitals were purposively selected because they manage high volumes of deliveries (between 17 and 50 per day in 2018) and therefore see large numbers of PPH cases. In these hospitals, medical officers (providers who hold a medical degree) may provide supervision but it is typically nurse-midwives, and nursing students who provide care throughout labor and delivery*.*

### Participants

Qualified providers were sampled based on their availability and their involvement in another component of the overall PPH study from a roster of approximately 300 qualified health care workers involved in maternity care in the study facilities (a convenience sample). Students were sampled based on their availability when they were present in the facility (no roster was provided for this group). The sample included consultants, medical officers, clinical officers, nurse-midwives, and nurse-midwifery students across all three study hospitals. Consultants are fully trained medical doctor specialists such as obstetrician-gynecologists, surgeons or pediatricians with postgraduate training and medical officers are licensed medical doctors who have completed six years of undergraduate training. Clinical officers are non-physician clinicians who undergo three to four years of training (a diploma degree). Clinical officers receive less training than medical officers, have a more restricted scope of practice and are accredited and licensed. In this setting, however, qualified nurses are the primary caregivers.

### Measurement tool

An open-ended knowledge questionnaire was adapted from the USAID Maternal and Child Health Integrated Program (MCHIP) interview & knowledge test [30] (previously administered in Kenya as part of a large evaluation of the quality of maternity care in 2010–11) [31], the World Bank Kyrgyz Republic results-based financing evaluation health worker knowledge test [32], and a knowledge test used in a recent study of the quality of maternity care in health facilities in Uganda [14]. We extracted knowledge questions from previous tools and developed additional questions in the same open-ended style, with the goal of being comprehensive in our coverage of knowledge domains that are relevant to PPH care. Contextually relevant adaptions were made in accordance with the Kenyan national guidelines for quality obstetrics and perinatal care [33], informed by consultations with clinicians in Kenya and the United States.

The final questionnaire included questions on provider characteristics such as training and experience in maternity care, in addition to knowledge of maternal and newborn care. The knowledge component of the interview comprised 20 questions on protocols for delivery of care from admissions through discharge. Participants were asked to freely list the clinical actions that they would take in different scenarios. The questions were read aloud to the participant and their verbal responses were recorded on paper questionnaires. Enumerators were instructed not to prompt health providers on their responses. Interviews were conducted face-to-face in private areas in order to ensure the confidentiality of responses. Interviews lasted approximately an hour.

Our analysis focused on knowledge of technical clinical protocols for maternal care. We excluded eight questions related to interpersonal care (for example: “What are the times or situations when a health worker should explain to the woman and/or her companion what is happening?”) and to neonatal care. To measure knowledge in different domains, we classified questions into three categories which were informed by both prior groupings (such as the WHO Standards for improving Quality of Maternal and Newborn Care) and evidence from the literature [5, 15, 34–36]. Additional file 1 documents components of the main studies used. These domains were: risk assessment, prevention, and management. These domains correspond with different phases of care (with risk assessment done before delivery; prevention done around the time of delivery; and management done when emergencies occur). The risk assessment domain included questions about what to check for in a patient’s admission history when admitted, and routine monitoring that should be carried out during labor. The prevention domain included questions on basic equipment that should be prepared before delivery, immediate maternal care after delivery, PPH prevention protocols, and appropriate counseling that should be given prior to discharge such as making patients aware of various danger signs (e.g., difficulty emptying the bladder). Lastly, the management domain included actions that are appropriate for women who present with PPH. All questions from the assessment and the correct responses are shown in Additional file 2.

### Statistical methods

We first described the characteristics of the providers in the study sample, including provider cadres, education level, age, gender, work experience, and participation in in-service training on PPH or Basic Emergency Obstetric and Newborn Care (BEmONC). While we are unable to speak to the specific types of training received at each facility, PPH training typically includes training on the prevention and management of PPH. These tend to be general and are rarely comprehensive. BEmONC training covers the necessary skills for handling obstetric emergencies such as postpartum infection, pre-eclampsia/eclampsia, postpartum hemorrhage, essential newborn care and resuscitation*.* Second, we analyzed provider responses to each of the included survey questions. We scored the providers’ responses to each question by dividing the total number of correct actions that a provider mentioned by the total number of recommended actions based on clinical guidelines. The possible score for each question ranged from zero to one, with zero indicating that the provider listed none of the recommended actions and one indicating that they listed all of the recommended actions. In this analysis, we included all of the recommended maternity-related clinical actions that providers should have mentioned, even if the actions were not specifically related to PPH. Given the important role that students play in the setting of this study, we compared the average score of each question for qualified health workers (i.e., nurses, clinical officers, medical officers, and consultants) to students. We estimated 95% confidence intervals around these scores using a normal approximation.

Third, we measured knowledge in each of the three domains of PPH care: PPH risk assessment, prevention, and management. Knowledge in each domain was defined as the sum of actions a provider mentioned for each domain divided by the total number of recommended actions in each domain. All clinical actions included in this analysis are bolded items in column three of the table in Additional file 2. There is some repetition in potential correct responses across questions about PPH management. For example, providers should have mentioned “administer a treatment uterotonic” in response to the questions about PPH from atonic uterus, PPH from retained placenta, and PPH due to lacerations. Details on how this is incorporated into scores can be found in Additional file 2.

Finally, we used Ordinary Least Squares (OLS) linear regression to examine characteristics associated with knowledge of PPH protocols. We ran separate models for each knowledge domain (assessment, prevention, and management). Associated characteristics included provider gender, age, education, specialization, years of experience in maternity care,^1^ participation in relevant in-service training, and self-reported closeness of relationships with colleagues. The closeness of providers’ relationships with their colleagues was measured using a survey question that asked providers to circle the picture that best represented their relationship with other providers showing four pictures ranging from A-D; option A showed separate circles representing distant working relationships, while option D showed overlapping circles representing “close” working relationships. This question was adapted from the Adapted Inclusion of Others in Self Scale [37] by Ashraf et al. (2016) [38] and is shown in Additional file 3. It was included as a proxy measure to explore the possibility of knowledge spillover from close peers, since maternity care in this setting is generally conducted by teams.

All regressions included facility and enumerator fixed effects and used robust standard errors. We defined statistical significance at the α = 0.05 level. All data were analysed using Stata, version 17.

### Sensitivity analyses

We tested several approaches to handling missing covariate data in our regression analysis. In our main models, we used multiple imputation in Stata to impute missing values of covariate data. We describe the details of our main approach and the alternate approaches we tested in Additional file 4.

It is also possible that using our measurement method, providers appeared more knowledgeable if they listed more clinical actions, even if they listed unnecessary or harmful actions (since we do not take away points for these additional actions). To assess the sensitivity of our measurement approach to this issue, we evaluated the extent to which this changed knowledge scores. We identified providers who mentioned harmful actions such as asking a patient to walk shortly after PPH identification, conducting a laparotomy, or initiating breastfeeding whilst managing PPH. Harmful practices were informed by the Kenyan Guidelines [36]. We then tested whether the probability of mentioning a harmful action increased as a provider listed more actions. Lastly, we tested the robustness of our findings to logistic regression instead of linear regression.

## Results

### Provider sample

Table 1 provides a description of our sample. A total of 173 providers, including students, were interviewed. One incomplete interview was dropped, resulting in an analytic sample of 172. The average provider in in the sample was 29 years old with 3 years of experience working in maternity care. Providers were mostly female (72%). The majority of providers interviewed were qualified nurse-midwives (46%) or nurse-midwifery students (37%).^2^ Two-thirds (67%) of providers had received in-service training on PPH, most of which occurred within the past 6 months (89%). Only 45% of providers had ever participated in additional Basic Emergency Obstetric and Newborn Care (BemONC) training. One in every five providers had never participated in any of these forms of in-service training. While participation in in-service training varied by cadre, we did not find systematic patterns: for example, a larger portion of consultants, medical officers, and clinical officers had participated in BEmONC training than nurse-midwives; and a larger portion of qualified nurses had participated in in-service PPH training than other cadres.

**Table 1:** Sample characteristics

Characteristic	Mean
Age in years (*N* = 172)	29
Number of years worked in maternity care in entire career (*N* = 132)	3
Females (*N* = 172)	72%
Providers holding respective positions (*N* = 171)	
Consultant, medical or clinical officer	17%
Qualified nurse	47%
Nurse-midwifery student	37%
Specialist (*N* = 171)	22%
Participated in additional in-service PPH training (*N* = 170)	67%
Participated in additional in-service PPH training in the past 6 months (*N* = 112)	89%
Participated in additional in-service BEmONC training (*N* = 170)	45%
Participated in additional in-service BEmONC training in the past 6 months (*N* = 67)	73%

This table presents the provider characteristics across all three facilities. Overall, *N* = 172 providers were included in the analytic sample. The total number of providers who answered each question are in parentheses (N). In the second column, the mean is presented as a percent where appropriate

*PPH* Postpartum Hemorrhage training, *BEmONC* Basic Emergency Obstetric and Newborn Care training

### Overall knowledge scores

**Fig. 1:**
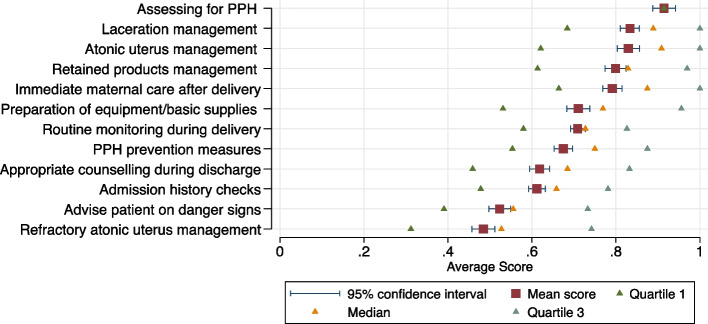
Knowledge of Routine care and PPH care (all actions), by question Notes. Figure 1 shows the average score for each question in the provider knowledge assessment from lowest to highest. Scores are calculated as the proportion of correct clinical actions that providers mention in response to open-ended questions. The y-axis shows the question. The x-axis shows the average score [0,1]. The red points indicate the average provider score for each question. The blue bars represent the 95% confidence interval around the average, estimated based on the assumption that scores are normally distributed. The triangles represent their respective quartiles

Figure 1 shows the average score and distribution of scores for each knowledge question. Providers scored an average of 0.71 (95% CI: 0.69–0.72) across all questions; this means that, on average, providers mentioned 71% of the recommended clinical actions. Providers scored lowest on the questions about the management of refractory PPH due to atonic uterus (average score of 0.48 out of 1.00; 95% CI: 0.46–0.51), danger signs to discuss with the mother prior to discharge (0.52; 95% CI: 0.50–0.51), admissions history checks (0.61; 95% CI 0.59–0.63) and appropriate counselling during discharge (0.62; 95% CI 0.59–0.64). The remainder of knowledge scores were all above 0.65 on average, with providers scoring the highest for questions relating to PPH management caused by lacerations (0.86; 95% CI 0.84–0.89), uterine atony (0.84; 95% CI 0.82–0.87), and retained placenta (0.81; 95% CI 0.78–0.83).

Table 2 shows that average scores generally did not differ between students and qualified health workers. However, qualified providers had slightly higher knowledge of immediate maternal care clinical protocols, such as monitoring vitals immediately after delivery (0.8 vs. 0.7; *p*-value < 0.01), and PPH assessment.

**Table 2:** Average knowledge scores for students and qualified providers

Question	Total (SD)	Qualified provider (SD)	Student (SD)	*P*-value
Q1 Admissions History	0.6 (0.1)	0.63 (0.1)	0.59 (0.1)	0.09
Q2 Routine observations/monitoring during delivery	0.7 (0.1)	0.72 (0.1)	0.69 (0.1)	0.1
Q3 Basic equipment/supplies available in delivery room	0.7 (0.2)	0.7 (0.2)	0.72 (0.2)	0.36
Q4 Immediate maternal care or health checks	0.8 (0.2)	0.82 (0.1)	0.73 (0.2)	< 0.001
Q5 PPH Prevention actions	0.7 (0.1)	0.67 (0.1)	0.68 (0.2)	0.82
Q6 Assessing/diagnosing PPH	0.9 (0.2)	0.94 (0.1)	0.87 (0.2)	0.02
Q7 PPH Management: Atonic Uterus	0.8 (0.2)	0.82 (0.2)	0.82 (0.2)	0.56
Q8 PPH Management: Retained placenta/products	0.8 (0.2)	0.81 (0.2)	0.79 (0.2)	0.46
Q9 PPH Management: Lacerations/Tears	0.8 (0.1)	0.78 (0.1)	0.79 (0.2)	0.86
Q10 PPH Management: Atonic Uterus, refractory	0.5 (0.2)	0.48 (0.2)	0.48 (0.2)	1.00
Q11 Topics to discuss prior to discharge	0.6 (0.2)	0.61 (0.2)	0.63 (0.1)	0.56
Q12 Awareness of danger signs	0.5 (0.2)	0.51 (0.2)	0.55 (0.2)	0.21

This figure shows the mean score for each question in the provider knowledge assessment by student (*N* = 63) or qualified provider. (*N* = 109). Provider questions are listed in Additional file 2. Scores are calculated as the proportion of correct clinical actions that providers mention in response to open-ended questions. Standard deviations in parentheses. *P*-values are presented for t-tests comparing the mean knowledge level among qualified health care providers with the mean level among students

### Knowledge of PPH risk assessment, prevention, and management

**Fig. 2:**
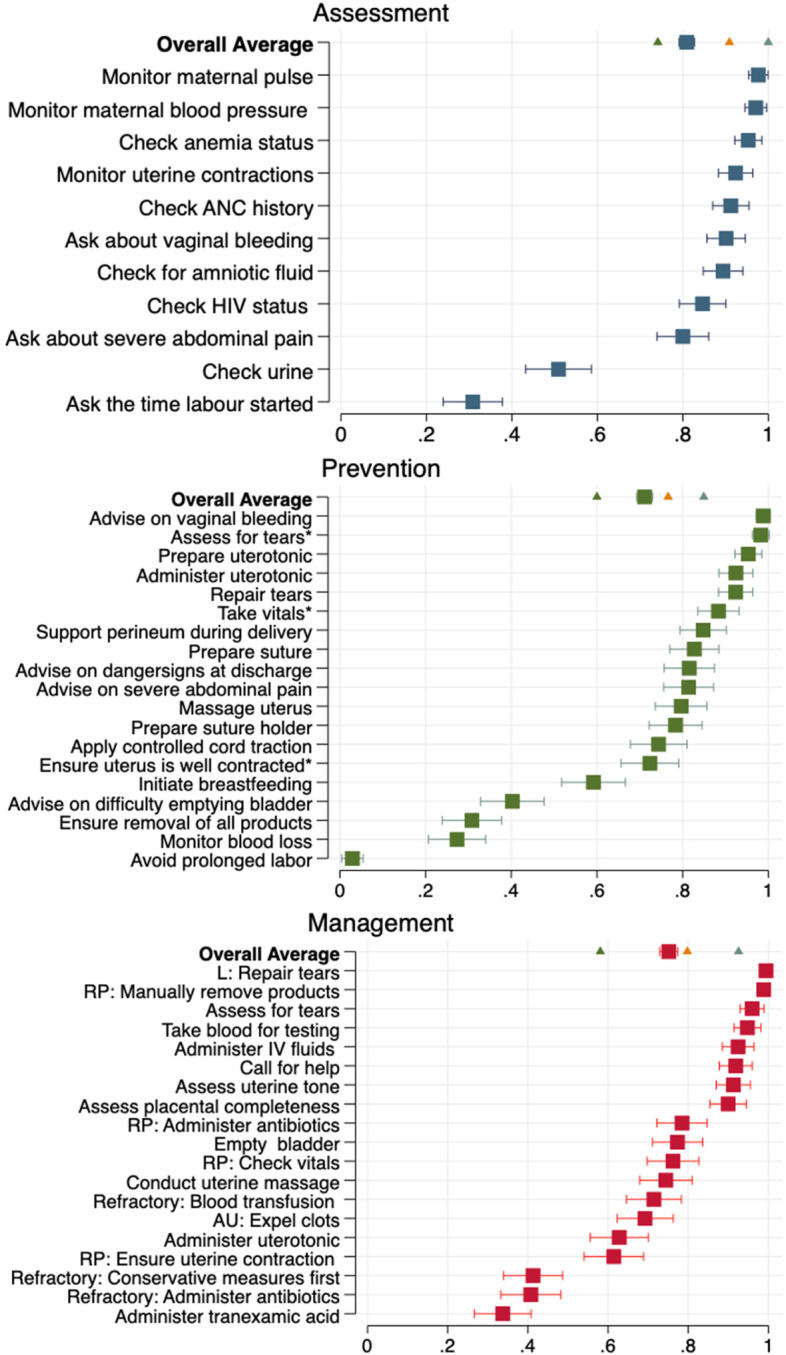
Proportion of providers that mention each action by domain and the overall average score Notes. This figure is sorted by the proportion of providers that mention each action. Actions marked with an asterisk under “Prevention” take time into consideration and should be done immediately or within the first hour of delivery. Under PPH management, common actions that should be taken for all primary PPH cases are calculated as “mentioned” if a provider mentions it at least twice throughout the interview for any question relating to the management of primary PPH. For example, “call for help”. Other actions undermanagement are specific to a cause – AU: Uterine Atony; RP: Retained Products; L: Lacerations and Refractory: Refractory Uterine Atony

Figure 2 shows average scores for the three PPH-related knowledge domains: risk assessment, prevention, and management.^3^ Providers scored the highest overall for risk assessment (average scores of 0.81 out of 1.00; 95% CI 0.79–0.83). That is, on average providers mentioned 81% of clinical actions included in this domain. In comparison, providers scored an average of 0.71. (95% CI 0.69–0.73) for preventive measures and 0.75 (95% CI 0.73–0.77) for knowledge of PPH management protocols.

The largest gaps in knowledge of risk assessment protocols were in checking the time when labor started (31% of providers mentioned this action; 95% CI 0.24–0.37) and monitoring urine output (51%; 95% CI 0.43–0.57). Almost all providers mentioned checking anemia status (95%; 95% CI 0.92–0.99), and monitoring maternal blood pressure (97%; 95% CI 0.94–0.99) and pulse (98%; 95% CI 0.95–1.00) during labor.

The largest gaps in knowledge of preventive care protocols were in avoiding prolonged labor (3% of providers mentioned this; 95% CI 0.01–0.05), monitoring blood loss (27%; 95% CI 0.21–0.34), ensuring the removal of all products (31%; 95% CI 0.24–0.38), and advising a mother to return to the emergency ward if she has difficulty emptying her bladder (40%; 95% CI 0.32–0.47). A large majority of providers mentioned advising the patient to return to the emergency ward in the case of severe vaginal bleeding (98%; 95% CI 0.88–1.00), checking for (92%; 95% CI 0.88–0.96), and repairing tears (98%; 95% CI 0.96–1.00), and preparing (95%; 95% CI 0.92–0.99) and administering a uterotonic (92%; 95% CI 0.89–0.96).

Finally, the majority of providers mentioned all of the recommended PPH management actions, with three exceptions. In the management of refractory PPH, only 41% of providers (95% CI:0.34–0.48) correctly listed conservative measures before more invasive measures, and only 41% of providers (95% CI: 0.33–0.48) mentioned the need to administer antibiotics. Only 34% of providers mentioned administering tranexamic acid in at least three of the relevant PPH cases described in the interview (95% CI 0.27–0.41).

### Factors associated with knowledge of PPH protocols

Table 3 provides information on the variables that had information missing. The years of experience variable had the largest percentage of missing data (17% of the sample). The remainder of the variables had missing data on less than 3% of the sample.

**Table 3:** Missing data

Variable	Observations missing	Percent Missing
Provider age in years	6	3%
Experience in years	29	17%
Whether the provider is a specialist	1	1%
Provider position	1	1%
Relationship with peers	5	3%
Additional in-service BEmONC training	2	1%
Additional in-service PPH training	2	1%
Additional in-service UBT training	2	1%

Results from the linear regression models are presented in Table 4. In our regression analysis, a few of the provider characteristics were statistically significantly associated with knowledge of protocols. For knowledge of assessment protocols, we found that relative to consultants and medical officers, being a nurse was associated with significantly higher scores in the multiple imputation model (OLS coefficient of 0.08; 95% CI: 0.02–0.13). For knowledge of prevention protocols, we found that experience in years was not associated with higher scores and had a coefficient of 0.00 (95% CI: 0.00-0.08). However, having a specialization was associated with higher knowledge scores compared to health providers who did not specialize (0.05; 95% CI: 0.00-0.09). We found that being female (0.04; 95% CI: 0.006-0.08) and having a bachelor’s degree (0.05; 95% CI: 0.01-0.08) was associated with higher knowledge of PPH management protocols. Having a master’s degree (-0.10; 95% CI: -0.18-0.01) and any BEmONC training (-0.05; 95% CI: -0.09—-0.005) was negatively associated with PPH management scores. In addition, reporting a closer relationship with colleagues was statistically significantly associated with higher knowledge of PPH management protocols (0.16; 95% CI: 0.05-0.26).

**Table 4:** Multivariate OLS regressions

	**Assessment**	**Prevention**	**Management**
Female gender	-0.02	0.03	0.04**
	(0.02)	(0.02)	(0.02)
Age in years	-0.00	-0.00	0.00
	(0.00)	(0.00)	(0.00)
Education: Bachelors (Ref = < Bachelors)	0.00	-0.01	0.05**
	(0.02)	(0.02)	(0.02)
Education: Masters	-0.08	-0.02	-0.09**
	(0.09)	(0.04)	(0.04)
Experience (years)	0.00	0.00**	0.00
	(0.00)	(0.00)	(0.00)
Specialisation (Reference = No)	-0.01	0.05**	0.01
	(0.03)	(0.02)	(0.03)
Position: Qualified nurse (Reference = Consultants)	0.08**	0.02	0.03
	(0.03)	(0.03)	(0.03)
Position: Student (Reference = Consultants)	0.04	0.02	0.01
	(0.03)	(0.03)	(0.04)
Relationship with peers: C (Ref = B)	-0.05	0.02	0.15***
	(0.03)	(0.05)	(0.06)
Relationship with peers: D (Ref = B)	-0.05	0.04	0.15***
	(0.03)	(0.05)	(0.06)
Additional in-service BEmONC training	0.03	-0.01	-0.05**
	(0.02)	(0.02)	(0.02)
Additional in-service PPH training	-0.01	0.00	0.05*
	(0.02)	(0.02)	(0.03)
Additional in-service UBT training	0.03	-0.01	-0.01
	(0.02)	(0.02)	(0.02)
Constant	0.83***	0.68***	0.51***
	(0.06)	(0.06)	(0.08)
Observations	172	172	172

All models include enumerator fixed effects and facility fixed effects. In Kenya, a bachelor's degree for medicine is a 6-year degree program. A bachelor’s degree in nursing is a 5 years program unlike other typical 4 year undergraduate programs, and a master's degree in medicine is a postgraduate-level degree (typically 3 years full-time); medics undertaking the program are Registrars

^***^p < 0.01, ** *p* < 0.05, * *p* < 0.1 Robust standard errors in parentheses

### Sensitivity analyses

We found that our main conclusions did not change when our analysis accounted for the fact that some providers listed more clinical actions or harmful actions in response to open-ended knowledge questions. Few providers mentioned harmful actions for prevention and the management of PPH caused by lacerations, or the management of refractory PPH caused by uterine atony. A greater proportion of providers mentioned a harmful action for initial treatment of PPH caused by uterine atony: examples included uterine packing, initiating breastfeeding, or conducting a laparotomy in theatre.^4^

Associations between knowledge domains and covariates were largely consistent across all sensitivity analyses, giving confidence to the main findings. There were some small changes in our results subsequent to several different sensitivity analyses. In the complete case model, being a nurse was not significantly associated with knowledge of assessment protocols. Having a specialization was associated with higher knowledge of prevention protocols, but this was not statistically significant. Using logistic regression, we found that although coefficients were directionally similar, in some cases precision was lower. Complete case analysis (*n* = 129), mean imputation, and logistic regression results can be found in Additional file 4.

## Discussion

We analysed health care providers’ knowledge of PPH clinical protocols using data from comprehensive in-person knowledge assessments in three referral hospitals in Kenya. We found significant knowledge gaps across three domains of knowledge: knowledge of risk assessment protocols, preventive care protocols, and PPH management protocols. Providers were least knowledgeable of preventive care protocols and most knowledgeable of PPH management protocols. Within PPH management protocols, providers were more knowledgeable of protocols for the management of primary PPH than of refractory PPH. Finally, in-service training was not associated with higher knowledge scores, but gender, education, provider cadre, and closer relationships with colleagues were positively associated with knowledge.

Our results are consistent with literature from Nigeria and Tanzania that found providers less knowledgeable about PPH prevention, but able to recall steps in the management of PPH [18, 20]. In Tanzania and Ethiopia, only 9% and 38% of study participants were knowledgeable about AMSTL (an important component of PPH prevention), respectively [8, 39]. A 2010 study in Kenya using the Maternal and Child Health Integrated Program (MCHIP) survey tool on a nationally representative sample of senior staff found that the majority of health workers interviewed were knowledgeable of certain actions such as administration of uterotonics (83% of providers) and massaging the uterus (79%). Overall knowledge of PPH management protocols was low (42%), specifically on actions such as emptying the bladder [31]. Our results, which come a decade later than the previous study, demonstrated higher average scores for PPH management. However, it is not clear whether this indicates improvements over time because the MCHIP sample differed from our sample in important ways: our study was in a purposive sample of three referral hospitals, while MCHIP was in a nationally representative sample of health facilities, and our sample included all cadres while MCHIP surveys were conducted with members of more senior cadres.

We found that average knowledge was exceptionally high (close to 100%) for some actions, but low (under 10%) for others. One reason for this could be that some actions were more salient, or that they were perceived to be more critical than others. Understanding why certain actions were more salient than others may be important for future research, particularly when comparing knowledge to quality of care observed.

Past studies from Tanzania and Uganda have found that years of experience does not significantly predict knowledge [20], but provider cadre (being a clinical/nursing officer) does [14]. In Tanzania, two further studies showed professional qualification as an important factor in managing PPH [8, 10] In our study, qualified nurses had higher average knowledge scores than consultants and medical or clinical officers. Since it is typically nurses who deliver care, this may be a result of learning by doing. Strikingly, students had very similar knowledge scores to qualified nurses, despite their lack of experience; this could indicate that knowledge assessments like the one used in this study measure book knowledge more than knowledge gained from experience.

Our findings on in-service training have important implications for quality improvement efforts. We found that participation in in-service training was not associated with higher knowledge scores. This is in line with evidence from a wide range of countries in sub-Saharan Africa that in-service training does not improve the quality of care [21]. However, when asked, “Among the various things related to your working situation that you would like to see improved, can you tell me the things that you think would most improve your ability to provide good quality care services?”, 95% of providers in our sample mentioned they would like additional training and 74% mentioned that they would like more supportive supervision.^5^ Those who said that they did not want more training or more supervision tended to have lower knowledge scores. It is possible that in-service training approaches and opportunities to learn need to be better designed [17, 40]. For example, the use of didactic teaching methods typical in low resource settings has less focus on the development of critical thinking skills and the hands-on skills needed to assess and manage patients [17, 40]. However, past studies in Ethiopia and Uganda have found that training is positively associated with knowledge [8, 14], but knowledge may fade as time passes after training sessions.

Our findings point to possible opportunities for improvement efforts. We found that providers who reported close relationships with their colleagues had higher average knowledge scores. While a thorough exploration of this topic was beyond the scope of our study, it is possible that this finding points to a role for knowledge spillover within teams or for positive effects of collaborative team environments on quality of care. To date, the literature on clinical knowledge has analyzed individual provider knowledge levels, however, team-based analyses may be an important future research direction for understanding how peer learning may improve provider knowledge and quality of care [41–43]. For example, future studies could examine how team members learn from each other or how the knowledge of different individual team members contributes to overall team performance. Second, we found significant variation across facilities, which persisted after adjusting for differences in the composition of our sample (in terms of health care provider cadres) across facilities. This could be driven by the selection of providers into health facilities (e.g., based on provider preferences, or approaches to recruitment), or by facility-level factors. This suggests that various factors such as facility norms, supervision, and mentorship. Future research could further focus on understanding differences across facilities to inform targeted programming to improve health worker knowledge.

This study has several limitations. Firstly, there is variation in the literature in how clinical knowledge is measured. Therefore knowledge scores and proportions across studies may not be directly comparable. Secondly, this study used a convenience sample of health care providers. There may have been differences in the types of providers who provided consent and were available for interview. However, we aimed to achieve representativeness by offering to schedule interviews flexibly in alignment with providers’ schedules. Thirdly, this study did not directly match providers’ knowledge of clinical protocols to their actual adherence. There may have been differences between these in practice, as suggested by a growing literature on know-do gaps [14]. However, since peer learning may be an important factor associated with knowledge, examination of both knowledge and adherence at the team level is critical for future research.

## Conclusion

In summary, we found important gaps in provider knowledge of clinical protocols for PPH care in referral hospitals in Kenya, where many emergency obstetric cases are managed. Participation in relevant in-service training was not a significant predictor of knowledge. There is a clear need for innovations in clinical training to ensure that providers in teaching referral hospitals are prepared to prevent, assess and manage PPH.

## Supplementary Information

**Additional file 1. **Sources for classifying domains.

**Additional file 2. **Measurement of PPH knowledge scores.

**Additional file 3. **Self-reported relationship with colleagues.

**Additional file 4. **Sensitivity analyses.

**Additional file 5. **Distribution of interviewed cadre across facilities.

**Additional file 6. **Facility-level comparisons of scores for each domain. 

**Additional file 7.** Differences in scores by domain across desired improvements.

## Data Availability

The datasets used and/or analysed during the current study are available from the corresponding author on reasonable request.

## Abbreviations

AMSTL: Active Management of the Third Stage of Labor
BEmONC: Basic Emergency Obstetric and Newborn Care
MCHIP: Maternal and Child Health Integrated Program
PPH: Postpartum Hemorrhage
SSA: Sub-Saharan Africa
USAID: United States Agency for International Development

## Footnotes

1 We include both years of experience in maternity care and age due to the high number of initial missing cases in the years of experience variable. Therefore, age can, to some extent, act as a proxy.

2 Additional file 5 presents the percentages of interviewed cadre within each facility. Differences are not statistically significant. However, a slightly higher portion of qualified nurses were interviewed in facility three relative to other facilities.

3 Facility-level comparisons for each domain can be found in the Additional file 6.

4 Details on harmful actions are provided in Additional file 4.

5 These results are shown in Additional file 7.
