# Supplementary material for: Health care providers’ knowledge of clinical protocols for postpartum hemorrhage care in Kenya: a cross-sectional study
Source: BMC Pregnancy Childbirth. 2022 Nov 10;22:828. doi: 10.1186/s12884-022-05128-6 (PMC9647972; doi:10.1186/s12884-022-05128-6)
Supplement: Supplementary file 1 — Additional file 1. Sources for classifying domains. [file 12884_2022_5128_MOESM1_ESM.pdf]

Additional File 1: Health care provider knowledge of clinical protocols for postpartum hemorrhage care in Kenya

Sources for grouping of factors

Tripathi et al., 2015[1]	Interpersonal *(not in scope of this study)*<br>Screening & monitoring<br>Infection prevention/control
Standards For Improving Quality Of Maternal And Newborn Care In Health Facilities (WHO, 2016)[2]	Routine care<br>Early diagnosis of complications<br>Appropriate management of complications
The Kenyan Maternal and New-born Health model (Ministry of Medical Services, & Ministry of Public Health and Sanitation Kenya, 2012[3]	Pre-pregnancy care *(unobserved in this study)*<br>Focused antenatal care<br>Essential obstetric care<br>Essential newborn care (*unobserved in this study)*<br>Targeted postpartum care<br>Post abortion care *(unobserved in this study).*
Nsangamay & Mash, 2019[4]	Active Management of the Third Stage of Labour (*importance of prevention*)

References

1. Tripathi, V., Stanton, C., Strobino, D., & Bartlett, L. (2015). Development and Validation of an Index to Measure the Quality of Facility-Based Labor and Delivery Care Processes in Sub-Saharan Africa. *PLOS ONE*, *10*(6), e0129491. https://doi.org/10.1371/journal.pone.0129491
2. WHO. (2016). *STANDARDS FOR IMPROVING QUALITY OF MATERNAL AND NEWBORN CARE IN HEALTH FACILITIES*. World Health Organization. https://www.who.int/docs/default-source/mca-documents/advisory-groups/quality-of-care/standards-for-improving-quality-of-maternal-and-newborn-care-in-health-facilities.pdf
3. Minisitry of Public Health and Sanitation Kenya. (2012). *National Guidelines for Quality Obstetrics and Perinatal Care*. Ministry of Medical Services & Minisitry of Public Health and Sanitation Kenya. http://guidelines.health.go.ke:8000/media/National_Guidelines_for_Quality_Obstetrics_and_Perinatal_Care. pdf
4. Nsangamay, T., & Mash, R. (2019). How to improve the quality of care for women with postpartum haemorrhage at Onandjokwe Hospital, Namibia: Quality improvement study. *BMC Pregnancy and Childbirth*, *19*(1), 489. https://doi.org/10.1186/s12884-019-2635-6
