# Supplementary material for: Health care providers’ knowledge of clinical protocols for postpartum hemorrhage care in Kenya: a cross-sectional study
Source: BMC Pregnancy Childbirth. 2022 Nov 10;22:828. doi: 10.1186/s12884-022-05128-6 (PMC9647972; doi:10.1186/s12884-022-05128-6)
Supplement: Supplementary file 2 — Additional file 2. Measurement of PPH knowledge scores. [file 12884_2022_5128_MOESM2_ESM.pdf]

Health care provider knowledge of clinical protocols for postpartum hemorrhage care in Kenya

Additional File 2: Measurement of PPH knowledge scores

Our analysis focused on knowledge of technical clinical protocols for maternal care. We excluded nine questions related to interpersonal care and to neonatal care. To measure knowledge in different domains, we classified questions into three categories: risk assessment, prevention, and management. The risk assessment domain included questions about what to check for in a patient's history when admitted, and routine monitoring that should be carried out during labor. The prevention domain included questions on basic equipment that should be prepared before delivery, immediate maternal care after delivery and PPH prevention protocols, and appropriate counseling that should be given prior to discharge such as making patients aware of various danger signs (e.g., difficulty emptying the bladder). Lastly, the management domain included actions that are appropriate for women who present with PPH. All questions from the assessment and the correct responses are shown in the table below.

There is an overlap in responses for questions relating to the immediate care of the mother after delivery (Q4) and the prevention of PPH (Q5). For these actions (massaging the uterus, assessing placental completeness/ensuring the removal of all products, and administering a uterotonic) we credit providers if they mentioned actions in both cases.

Additionally, for PPH management there are common actions that should be adhered to across various causes of PPH. Such actions are calling for help, administering a uterotonic, emptying the patient's bladder, massaging the uterus, administering tranexamic acid, sending blood for cross-matching, and administering IV fluids. For these actions, we credit providers if they mentioned these actions for PPH management in at least three (out of five possible times) throughout the interview on questions that ask about PPH management. A sensitivity analysis reveals that scores are similar if a similar process is followed crediting providers if they mention each action *at least once.* However, scores are statistically different when requiring providers to mention the action in *all* (five) cases. This includes common actions that should first be repeated in the case of refractory uterine atony management. Actions that should be taken when managing a specific cause of PPH such as repairing tears during managing PPH caused by lacerations were kept separate. We exclude less conservative measures that may be taken in cases relating to refractory PPH management since these actions vary on a case-by-case basis. For example, a less conservative measure such as a hysterectomy may not be necessary in all cases.

Questions asked during provider interview and responses

**Survey / Tool**	**Actions included in the questionnaire:**	**Actions included in knowledge analysis:**
**Q1: When a woman is admitted for labour and delivery, what should her provider ask about (or check in her ANC book) during her admission history?**	Last menstrual period	Last menstrual period
	ANC history	**ANC history**
	Referral information	Referral information
	Headaches/blurred vision	Headaches/blurred vision
	Vaginal bleeding	**Vaginal bleeding**
	Fever	Fever
	Swollen face or hands	Swollen face or hands
	Convulsions or loss of consciousness	Convulsions or loss of consciousness

	Severe difficulty breathing	Severe difficulty breathing
	Persistent cough	Persistent cough
	Severe abdominal pain	**Severe abdominal pain**
	Felt a decrease or stop in fetal movement	Felt a decrease or stop in fetal movement
	Anaemia status	**Anaemia status**
	HIV Status	**HIV Status**
		***Time labour started**
		*Previous medication taken
**Q2: Please tell me, when conducting a delivery, what routine observations or monitoring should be carried out during labor? Assume cephalic presentation.**	Monitor fetal heart rate	Monitor fetal heart rate
	Assess degree of molding	Assess degree of molding
	Assess cervical dilation	Assess cervical dilation
	Assess descent of head	Assess descent of head
	Monitor uterine contractions	**Monitor uterine contractions**
	Monitor maternal blood pressure	**Monitor maternal blood pressure**
	Monitor maternal pulse	**Monitor maternal pulse**
	Check the urine	**Check the urine**
	Check for amniotic fluid	**Check for amniotic fluid**
		*Monitor hydration
		*Check cord presentation
**Q3: What basic equipment and supplies must be available in the delivery room to ensure the mother and baby receives appropriate immediate care after birth?**	A complete delivery set	A complete delivery set
	Dry warm towels or cloths	Dry warm towels or cloths
	Sterile blade or scissors	Sterile blade or scissors
	Sterile or disposable cord ties / clamps	Sterile or disposable cord ties / clamps
	Cap for baby	*Excluded (relates to newborn)*
	Source of warmth: heating lamp or Incubator	
	Self-inflating ventilation	
	Newborn face mask size 0	
	Newborn face mask size 1	
	Mucus extractor/simple suction/ bulb Syringe	
	Flat surface	Flat surface
	Clock or watch with seconds	Clock or watch with seconds
	Uterotonic drug	**Uterotonic drug**
	Foley catheter	Foley catheter
	Suture holder	**Suture holder**
	Suture	**Suture**
	Antiseptic solution to wash the vulva	Antiseptic solution to wash the vulva
	Uterine balloon tamponade	*Excluded*
	Dry the baby and wrap in warm cloth	*Excluded (relates to newborn)*
	Observe for color	
	Assign APGAR score	
	Provide thermal protection (skin-to-skin)	
	Apply antiseptic or other material to cord stump	
**Q4: After delivery of the newborn, can you tell me about the immediate care or health checks that**	Take mother's vital signs	**Take mother's vital signs**
	Administer uterotonic	**Administer uterotonic**
	Provide uterine massage	**Provide uterine massage**
	Check for tears or lacerations	**Check for tears or lacerations**

**should be given to the mother?**	Ensure uterus is well-contracted (palpate uterus 15 minutes after delivery of placenta)	**Ensure uterus is well-contracted (palpate uterus 15 minutes after delivery of placenta)**
	Assess completeness of placenta and membranes	**Assess completeness of placenta and membranes**
	Apply controlled cord traction	Apply controlled cord traction
**Q5: During labour and delivery, what steps can be taken to prevent PPH?**	Support perineum during delivery	**Support perineum during delivery**
	Provide prophylactic uterotonic	**Provide prophylactic uterotonic**
	Massage uterus	**Massage uterus**
	Repair tears	**Repair tears**
	Assist the mother to start breastfeeding within the first hour of delivery	**Assist the mother to start breastfeeding within the first hour of delivery**
	Ensure empty bladder	**Ensure empty bladder**
		***Avoid prolonged labour**
		***Ensure removal of all products**
		***Monitor blood loss**
**Q6: What immediate actions, diagnostic tests, or interventions are appropriate for a woman who presents with, or develops, heavy bleeding postpartum?**	Assess completeness of placenta and membranes	**Assess completeness of placenta and membranes**
	Assess for perineal and vaginal lacerations	**Assess for perineal and vaginal lacerations**
	Assess uterine tone	**Assess uterine tone**
	Call for help	**Call for help**
	Empty urinary bladder	**Empty urinary bladder**
	Massage uterine fundus	**Massage uterine fundus**
	Cross-match blood	**Cross-match blood**
	Initiate IV access	**Administer IV fluids**
	Provide saline	
	Provide tranexamic acid	**Provide tranexamic acid**
**Q7: What actions, diagnostic tests, or interventions are appropriate for a woman who presents with, or develops, heavy bleeding postpartum from atonic uterus?**	Call for help	**Call for help**
	Massage the fundus	**Massage the fundus**
	Empty urinary bladder	**Empty urinary bladder**
	Give uterotonics	**Give uterotonics**
	Cross-match blood	**Cross-match blood**
	Initiate IV access	**Administer IV fluids**
	Provide Saline	
	Provide tranexamic acid	**Provide tranexamic acid**
	Expel clots	**Expel clots**
	Assure the woman	*Excluded from analysis*
	Bimanual compression of uterus	*Excluded from analysis (not done in all cases)*
	Abdominal compression of aorta	
	Prepare operating theatre	
	Raise foot of the bed	*Excluded from analysis*
	Insert uterine balloon tamponade	*Excluded from analysis*
	Uterine packing	*Excluded from analysis (incorrect)*
	Hysterectomy	*Excluded from analysis (not done in all cases)*
**Q8: What actions, diagnostic tests, or interventions are appropriate for a woman who presents with, or develops, heavy bleeding postpartum from retained**	Reassure woman	*Excluded from analysis (non-technical)*
	Empty urinary bladder	**Empty urinary bladder**
	Repeat uterotonic	**Repeat uterotonic**
	Manually remove placenta/products	**Manually remove placenta/products**
	Give IV fluids	**Give IV fluids**
	Monitor vital signs for shock	**Monitor vital signs for shock**

**placenta / products of conception after delivery?**	Check contraction of uterus	**Check contraction of uterus**
	Massage fundus after removal	**Massage fundus after removal**
	Give antibiotics	**Give antibiotics**
	Take blood for grouping and crossmatching	**Take blood for grouping and crossmatching**
	Prepare for theatre if bleeding does not improve	Prepare for theatre if bleeding does not improve
	Call for help	**Call for help**
	Provide Tranexamic Acid	**Provide Tranexamic Acid**
**Q9: What immediate actions, diagnostic tests, or interventions are appropriate for a woman who presents with, or develops, heavy bleeding postpartum from lacerations after delivery?**	Pack the tear	*Excluded (incorrect to do)*
	Repair the tear	**Repair the tear**
	Administer tranexamic acid	**Administer tranexamic acid**
	Call for help	**Call for help**
	Empty urinary bladder	**Empty urinary bladder**
	Massage uterine fundus	**Massage uterine fundus**
	Cross-match blood	**Cross-match blood**
	Initiate IV access	**Administer IV fluids**
	Provide saline	
**Q10: A patient has PPH from atonic uterus. She has already received a treatment dose of uterotonic and conducted uterine massage.<br><br>What are the next interventions that she should be given to manage the blood loss?**	Repeat dose of oxytocin	**Repeat dose of oxytocin**
	Repeat dose of tranexamic acid	**Repeat dose of tranexamic acid**
	Give IV fluids	**Give IV fluids**
	Start antibiotics	**Start antibiotics**
	Empty bladder	**Empty bladder**
	Continuous uterine massage	**Continuous uterine massage**
	Aortic or bimanual compression	*Excluded (not done in all cases)*
	Blood transfusion	**Blood transfusion**
	Explore in theater	**Explore in theater**
	Prepare for emergency surgery	*Excluded (not done in all cases)*
	Uterine artery ligation	
	Sub-total hysterectomy	
	Total hysterectomy	
	UBT	*Excluded*
	NASG	*Excluded*
	Refer to another facility	*Excluded*
		**Order score**[+]
**Q11: What topics should a provider discuss with a mother before she is discharged postpartum?**	Danger signs for the mother	**Danger signs for the mother**
	Danger signs for the baby	Danger signs for the baby
	Return to fertility	Return to fertility
	Healthy timing and spacing of pregnancies	Healthy timing and spacing of pregnancies
	HIV testing	HIV testing
	Family planning options	Family planning options
	Maternal nutrition	Maternal nutrition
	Infant feeding	Infant feeding
		Postnatal visits
		*Cord care
		*Perineal care
**Q12: You are about to discharge a mother who had an uncomplicated vaginal delivery. In counselling her with regards to danger signs, what signs will you tell her to observe that indicate that she should go to the**	Mother: bleeding	**Mother: bleeding**
	Mother: severe abdominal pain	**Mother: severe abdominal pain**
	Mother: severe headache or visual disturbance	Mother: severe headache or visual disturbance
	Mother: breathing difficulty	Mother: breathing difficulty
	Mother: fever or chills	Mother: fever or chills
	Mother: difficulty emptying bladder	**Mother: difficulty emptying bladder**
	Mother: epigastric pain	Mother: epigastric pain

**health centre immediately – day or night – without delay?**		*Mother: Eclampsia
		*Mother: Swelling
	Baby: fast or difficult breathing	*Excluded from analysis (relates to newborn)*
	Baby: fever	
	Baby: unusually cold	
	Baby: stops feeding well	
	Baby: less active than normal	
	Baby: whole body becomes yellow	

Notes: Column 1 represents the question number used throughout this analysis. Column 2 represents actions included in the knowledge tool as correct responses. Column 3 shows the actions included in the knowledge score construction; additional items were included based on the Kenyan guidelines are marked with an asterisk (*). **Bolded** actions relate specifically to the assessment, prevention and management of PPH. These actions were included in the PPH index. [+]Generated an indicator for if less conservative measures such as "hysterectomy" was mentioned post conservative measures (i.e., after oxytocin, massage, empty bladder)
