# Supplementary material for: Health care providers’ knowledge of clinical protocols for postpartum hemorrhage care in Kenya: a cross-sectional study
Source: BMC Pregnancy Childbirth. 2022 Nov 10;22:828. doi: 10.1186/s12884-022-05128-6 (PMC9647972; doi:10.1186/s12884-022-05128-6)
Supplement: Supplementary file 3 — Additional file 3. Self-reported relationship with colleagues. [file 12884_2022_5128_MOESM3_ESM.pdf]

Additional File 3: Health care provider knowledge of clinical protocols for postpartum hemorrhage care in Kenya

Question on self-reported relationship with colleagues

No.	Question	Response
3.11a	Circle the picture that best represents your relationship with the other providers you work with in the facility.<br>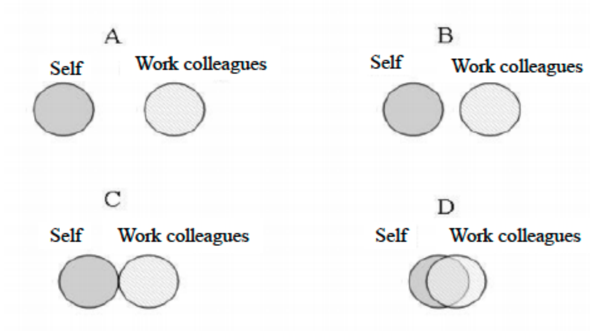	A……………………………1<br>B……………………………2<br>C……………………………3<br>D……………………………4

Notes: This image depicts the image that was shown to providers during the interview asking them to circle the picture that best represents their relationship with other providers in their facility.
