# Supplementary material for: Health care providers’ knowledge of clinical protocols for postpartum hemorrhage care in Kenya: a cross-sectional study
Source: BMC Pregnancy Childbirth. 2022 Nov 10;22:828. doi: 10.1186/s12884-022-05128-6 (PMC9647972; doi:10.1186/s12884-022-05128-6)
Supplement: Supplementary file 4 — Additional file 4. Sensitivity analyses. [file 12884_2022_5128_MOESM4_ESM.pdf]

Additional File 4: Health care provider knowledge of clinical protocols for postpartum

Sensitivity analyses

Harmful actions

While few providers mentioned harmful actions for prevention of PPH (N=1; 0.6 % of providers) and the management of PPH caused by lacerations (N=4; 2%), or retained placenta (N=4) (N=4; 2%), just under a third of providers mentioned a harmful action for PPH from uterine atony (N=51, 29 %) such as uterine packing, initiating breastfeeding, or conducting a laparotomy in theatre. Six providers mentioned harmful actions relating to the management of refractory uterine atony PPH such as initiating breastfeeding. The probability of mentioning a harmful action did not increase as a provider listed more actions.

Methods and results

For imputation analyses we relied on the assumption that data are missing at random conditional on the included variables1. Our multiple imputation models included all of the same variables as our main OLS models, including outcome variables although none of these values were missing. Coefficients and standard errors were adjusted according to Rubin's combination rule[1]. Binary and categorical variables were transformed into indicators and included in the model. We used 20 imputations and a joint multivariate normal distribution model. In sensitivity analyses, we used a complete case analysis (relying on the assumption that data were missing completely at random). In an additional model, we replaced missing values with the mean and included a missing data indicator for each variable. Tables A4-A6 present the regression results for complete case analysis, mean imputation, and logistic regression models. Post-estimation analysis of OLS regressions revealed that while the residuals of assessment were normally distributed (p=0.09), those of prevention (p=0.00) and management were not (p=0.00), offering strong evidence of non-linearity. We then analyzed a Generalised Linear Model (GLM). Since these results are largely consistent we focus on the OLS for ease of interpretation.

[1] To make this assumption we explored patterns of the missing data. We found that missing information on total years of experience does not differ significantly across age, gender, relation to peers or education and there was variation in these characteristics. Although all providers who had missing information on age were female (n=6), these providers were not missing information on total years of experience and other characteristics varied (such as position, relation to peers, training and education level). Additionally, outcome variables (knowledge scores across each domain) do not differ significantly between those with complete information and those with at least one variable missing.

Regression Results -- Assessment

	Complete Case analysis	Mean imputation	GLM
Gender (Reference = Male)	-0.02	-0.01	-0.11
	(0.02)	(0.02)	(0.13)
Age in years	0.02	0.02*	0.10
	(0.01)	(0.01)	(0.06)
Education: Bachelors (Ref= <Bachelors)	0.00	0.00	0.02
	(0.00)	(0.00)	(0.02)
Education: Masters	-0.01	-0.00	-0.06
	(0.02)	(0.02)	(0.12)
Experience (years)	-0.07	-0.08	-0.39
	(0.08)	(0.07)	(0.38)
Specialisation (Reference = No)	0.00	0.00	0.01
	(0.03)	(0.03)	(0.18)
Position: Qualified nurse (Reference = Consultants)	0.04	0.05	0.29
	(0.03)	(0.03)	(0.18)
Position: Student (Reference = Consultants)	0.03	0.05	0.21
	(0.03)	(0.03)	(0.20)
Relationship with peers: C (Ref = B)	-0.06*	-0.05	-0.41
	(0.03)	(0.03)	(0.26)
Relationship with peers : D	-0.05	-0.04	-0.38
	(0.03)	(0.03)	(0.26)
Additional in-service BeMONC training	0.03	0.03	0.14
	(0.02)	(0.02)	(0.10)
Additional in-service UBT training	0.02	-0.01	-0.06
	(0.02)	(0.02)	(0.13)
Additional in-service PPH training	-0.01	-0.01	0.16
	(0.02)	(0.02)	(0.12)
Facility 2 (Reference = Facility 1)	0.11***	0.11***	0.69***
	(0.03)	(0.03)	(0.20)
Facility 3 (Reference = Facility 1)	0.10***	0.10***	0.69***
	(0.02)	(0.02)	(0.12)
Enumerator 2 (Ref=1)	-0.10**	-0.11***	-0.58**
	(0.04)	(0.04)	(0.23)
Enumerator 3 (Ref=1)	-0.38***	-0.39***	-1.97***
	(0.06)	(0.05)	(0.34)
Enumerator 4 (Ref=1)	-0.06*	-0.07**	-0.37*
	(0.04)	(0.03)	(0.22)
Enumerator 5 (Ref=1)	-0.10*	-0.12**	-0.68*
	(0.06)	(0.05)	(0.37)
Enumerator 6 (Ref=1)	-0.18*	-0.19*	-1.15*
	(0.11)	(0.11)	(0.59)
Age in years (missing)		0.02	
		(0.03)	

Experience (years), (missing)		0.04	
		(0.04)	
Specialisation ; (missing)		0.06***	
		(0.02)	
Position; (missing)		0.12***	
		(0.03)	
Relationship with peers; (missing)		0.02	
		(0.04)	
BeMONC Training; (missing)		-0.21***	
		(0.04)	
Constant	0.59***	0.52***	0.06
	(0.18)	(0.18)	(1.13)
Observations	157	172	157
R-squared	0.32	0.38	

Robust standard errors in parentheses
*** p<0.01, ** p<0.05, * p<0.1

Regression Results – Prevention

	Complete Case analysis	Mean imputation	GLM
Gender (Reference = Male)	0.03	0.03	0.14*
	(0.02)	(0.02)	(0.09)
Age in years	-0.01	-0.01	-0.06
	(0.01)	(0.01)	(0.04)
Education: Bachelors (Ref= <Bachelors)	0.00	0.00	0.01
	(0.00)	(0.00)	(0.01)
Education: Masters	-0.02	-0.01	-0.11
	(0.02)	(0.02)	(0.09)
Experience (years)	-0.03	-0.03	-0.17
	(0.06)	(0.06)	(0.25)
Specialisation (Reference = No)	0.05*	0.05*	0.27**
	(0.03)	(0.03)	(0.13)
Position: Qualified nurse (Reference = Consultants)	0.02	0.02	0.07
	(0.03)	(0.03)	(0.14)
Position: Student (Reference = Consultants)	-0.01	0.00	-0.07
	(0.03)	(0.03)	(0.13)
Relationship with peers: C (Ref = B)	0.05	0.03	0.22
	(0.05)	(0.05)	(0.23)
Relationship with peers : D	0.07	0.05	0.32
	(0.05)	(0.05)	(0.24)
Additional in-service BeMONC training	-0.01	-0.01	-0.03
	(0.02)	(0.02)	(0.08)
Additional in-service UBT training	-0.01	-0.01	0.03
	(0.02)	(0.02)	(0.10)
Additional in-service PPH training	0.01	0.00	-0.06
	(0.02)	(0.02)	(0.08)
Facility 2 (Reference = Facility 1)	0.01	0.02	0.01
	(0.03)	(0.03)	(0.13)
Facility 3 (Reference = Facility 1)	0.16***	0.16***	0.80***
	(0.02)	(0.02)	(0.10)
Enumerator 2 (Ref=1)	-0.02	-0.02	-0.06
	(0.03)	(0.03)	(0.15)
Enumerator 3 (Ref=1)	-0.21***	-0.23***	-0.85***
	(0.05)	(0.05)	(0.24)
Enumerator 4 (Ref=1)	-0.10***	-0.10***	-0.45***
	(0.03)	(0.03)	(0.14)
Enumerator 5 (Ref=1)	-0.01	-0.02	-0.02
	(0.05)	(0.05)	(0.24)
Enumerator 6 (Ref=1)	-0.16**	-0.18***	-0.68**
	(0.07)	(0.07)	(0.30)
Age in years (missing)		0.03	
		(0.05)	
Experience (years), (missing)		0.06*	
		(0.03)	

Specialisation ; (missing)		0.35***	
		(0.02)	
Position; (missing)		0.10***	
		(0.02)	
Relationship with peers; (missing)		0.09*	
		(0.04)	
BeMONC Training; (missing)		-0.04	
		(0.07)	
Constant	0.83***	0.81***	1.55**
	(0.15)	(0.14)	(0.68)
Observations	157	172	157
R-squared	0.45	0.45	

Robust standard errors in parentheses
*** p<0.01, ** p<0.05, * p<0.1

Regression Results – Management

	Complete Case analysis	Mean imputation	GLM
Gender (Reference = Male)	0.04*	0.04**	0.20*
	(0.02)	(0.02)	(0.11)
Age in years	-0.00	-0.00	0.00
	(0.01)	(0.01)	(0.05)
Education: Bachelors (Ref= <Bachelors)	-0.00	-0.00	-0.01
	(0.00)	(0.00)	(0.02)
Education: Masters	0.04*	0.06***	0.20
	(0.02)	(0.02)	(0.13)
Experience (years)	-0.09**	-0.09*	-0.38*
	(0.04)	(0.05)	(0.21)
Specialisation (Reference = No)	0.00	0.01	0.04
	(0.03)	(0.03)	(0.15)
Position: Qualified nurse (Reference = Consultants)	0.05	0.04	0.19
	(0.03)	(0.03)	(0.15)
Position: Student (Reference = Consultants)	0.01	0.01	-0.02
	(0.04)	(0.04)	(0.20)
Relationship with peers: C (Ref = B)	0.19***	0.16***	1.04***
	(0.06)	(0.06)	(0.32)
Relationship with peers : D	0.19***	0.16***	1.00***
	(0.06)	(0.06)	(0.31)
Additional in-service BeMONC training	-0.06**	-0.05**	-0.29**
	(0.02)	(0.02)	(0.12)
Additional in-service UBT training	-0.01	-0.01	0.24*
	(0.02)	(0.02)	(0.13)
Additional in-service PPH training	0.04	0.05*	-0.09
	(0.03)	(0.03)	(0.11)
Facility 2 (Reference = Facility 1)	-0.07	-0.06	-0.49**
	(0.04)	(0.04)	(0.21)
Facility 3 (Reference = Facility 1)	0.15***	0.15***	0.91***
	(0.02)	(0.02)	(0.13)
Enumerator 2 (Ref=1)	0.04	0.05*	0.36**
	(0.03)	(0.03)	(0.16)
Enumerator 3 (Ref=1)	-0.20***	-0.21***	-0.69**
	(0.06)	(0.06)	(0.30)
Enumerator 4 (Ref=1)	-0.10***	-0.08***	-0.51***
	(0.03)	(0.03)	(0.14)
Enumerator 5 (Ref=1)	0.00	0.02	-0.02
	(0.06)	(0.06)	(0.30)
Enumerator 6 (Ref=1)	-0.23***	-0.24***	-0.80**
	(0.07)	(0.07)	(0.31)
Age in years (missing)		0.02	

		(0.07)	
Experience (years), (missing)		0.20***	
		(0.03)	
Specialisation ; (missing)		0.10***	
		(0.02)	
Position; (missing)		-0.08***	
		(0.03)	
Relationship with peers; (missing)		-0.01	
		(0.04)	
BeMONC Training; (missing)		0.04	
		(0.03)	
Constant	0.51***	0.54***	-0.24
	(0.18)	(0.16)	(0.95)
Observations	157	172	157
R-squared	0.57	0.55	

Robust standard errors in parentheses
*** p<0.01, ** p<0.05, * p<0.1

References

1. Rubin, D. B. (1996). Multiple Imputation after 18+ Years. *Journal of the American Statistical Association*, *91*(434), 473–489. https://doi.org/10.1080/01621459.1996.10476908
