# Supplementary material for: Health care providers’ knowledge of clinical protocols for postpartum hemorrhage care in Kenya: a cross-sectional study
Source: BMC Pregnancy Childbirth. 2022 Nov 10;22:828. doi: 10.1186/s12884-022-05128-6 (PMC9647972; doi:10.1186/s12884-022-05128-6)
Supplement: Supplementary file 5 — Additional file 5. Distribution of interviewed cadre across facilities. [file 12884_2022_5128_MOESM5_ESM.pdf]

Additional File 5: Health care provider knowledge of clinical protocols for postpartum hemorrhage care in Kenya

Distribution of interviewed cadre across facilities

	Total	Facility 1	Facility 2	Facility 3
Consultant, medical or clinical officer	28 (16%)	14 (16%)	8 (24%)	6 (11%)
Qualified nurse	79 (46%)	38 (44%)	14 (42%)	27 (51%)
Nursing-Midwifery student	63 (37%)	33 (38%)	11 (33%)	19 (36%)
Missing	2 (1%)	1 (1%)	0 (0%)	1 (2%)

Notes: This table shows the number and percentage (in parentheses) of cadre at each facility that is included in the present analytic sample. In total 172 providers were interviewed, comprising 86, 33, and 53 providers at Facility 1, 2, and 3 respectively.
