# Supplementary material for: Health care providers’ knowledge of clinical protocols for postpartum hemorrhage care in Kenya: a cross-sectional study
Source: BMC Pregnancy Childbirth. 2022 Nov 10;22:828. doi: 10.1186/s12884-022-05128-6 (PMC9647972; doi:10.1186/s12884-022-05128-6)

Additional file 6: Health care provider knowledge of clinical protocols for postpartum hemorrhage care in Kenya

Assessment scores

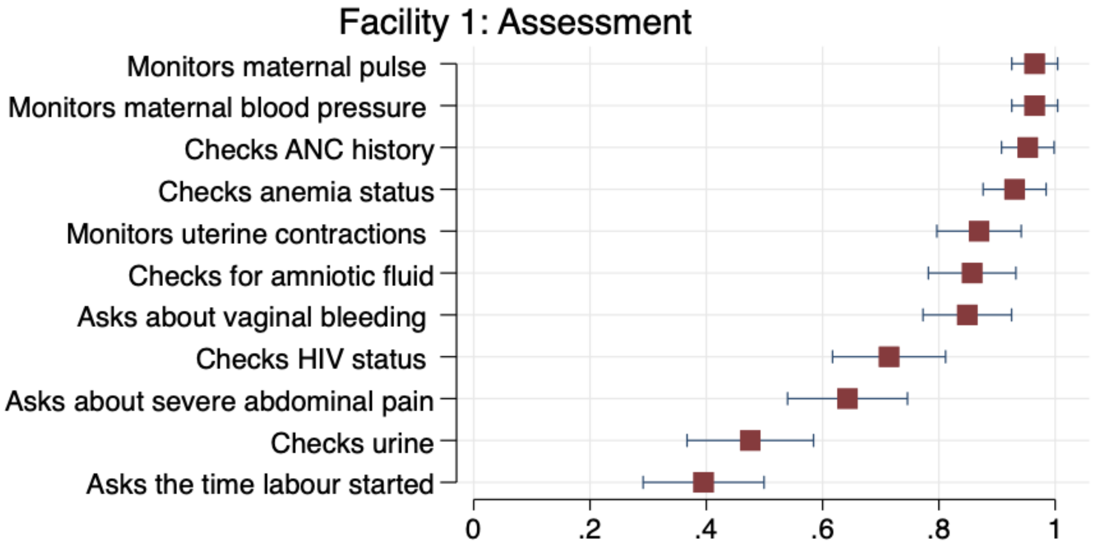

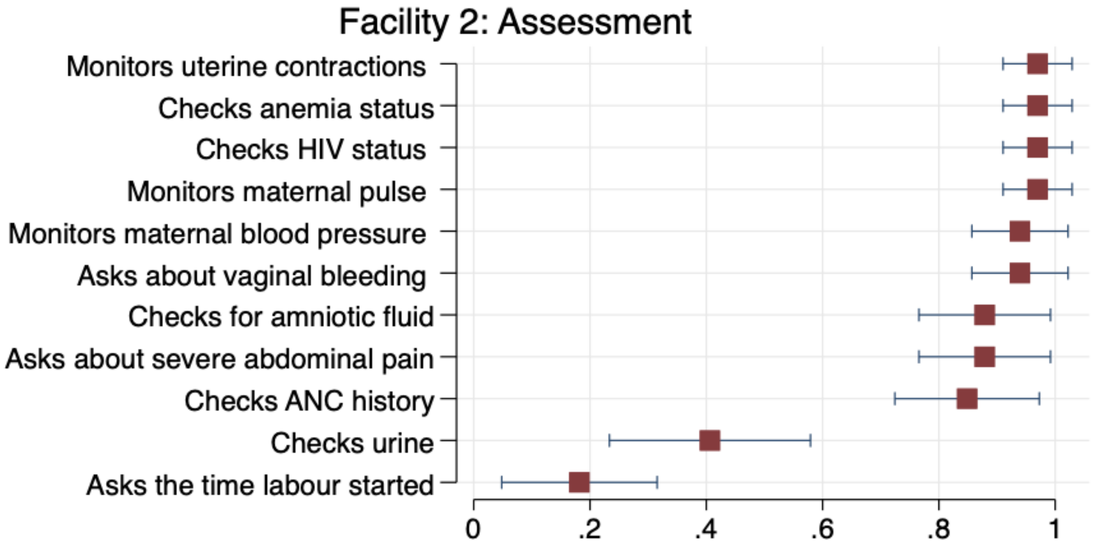

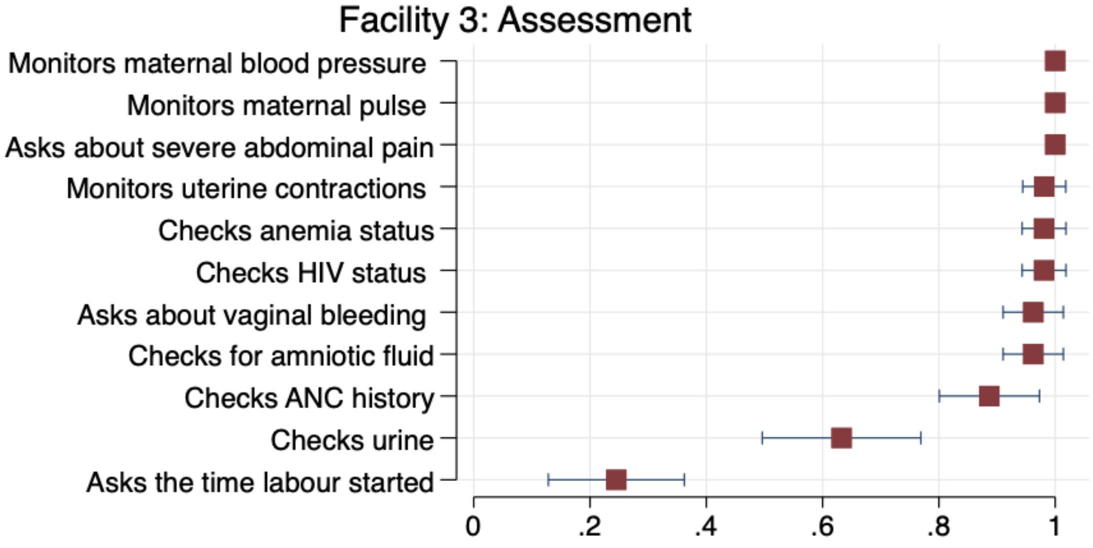

Prevention scores

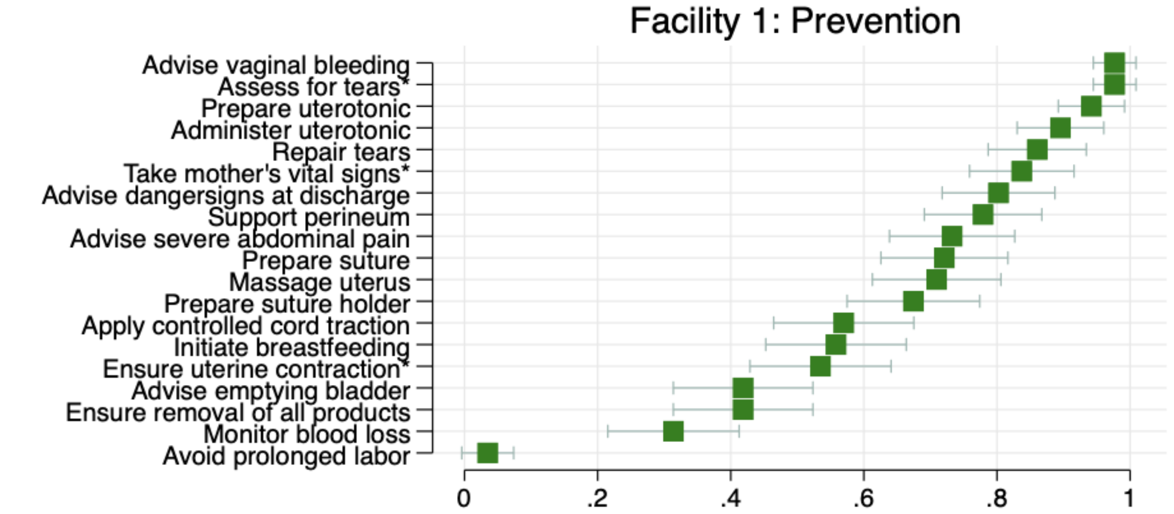

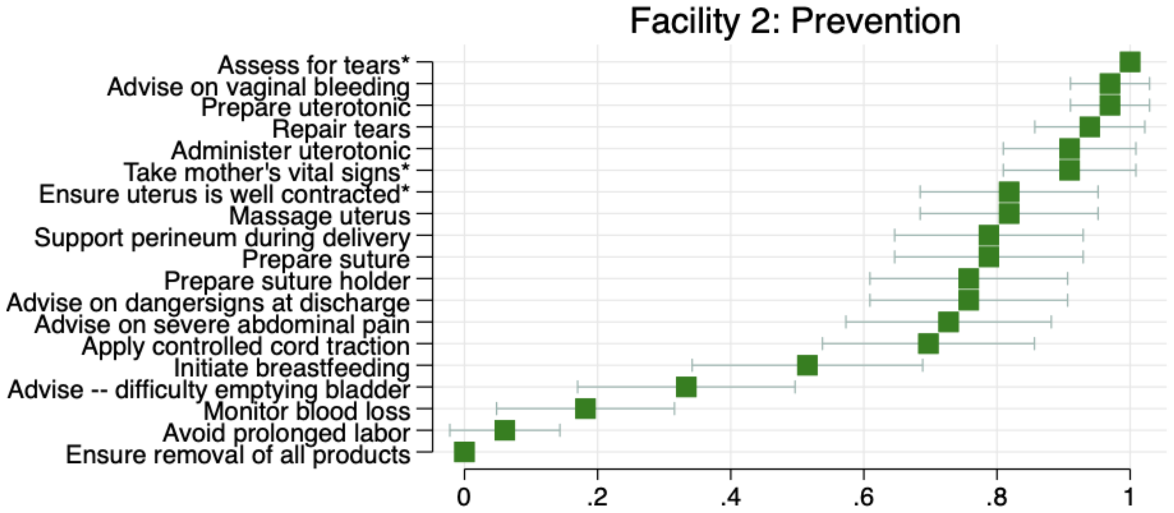

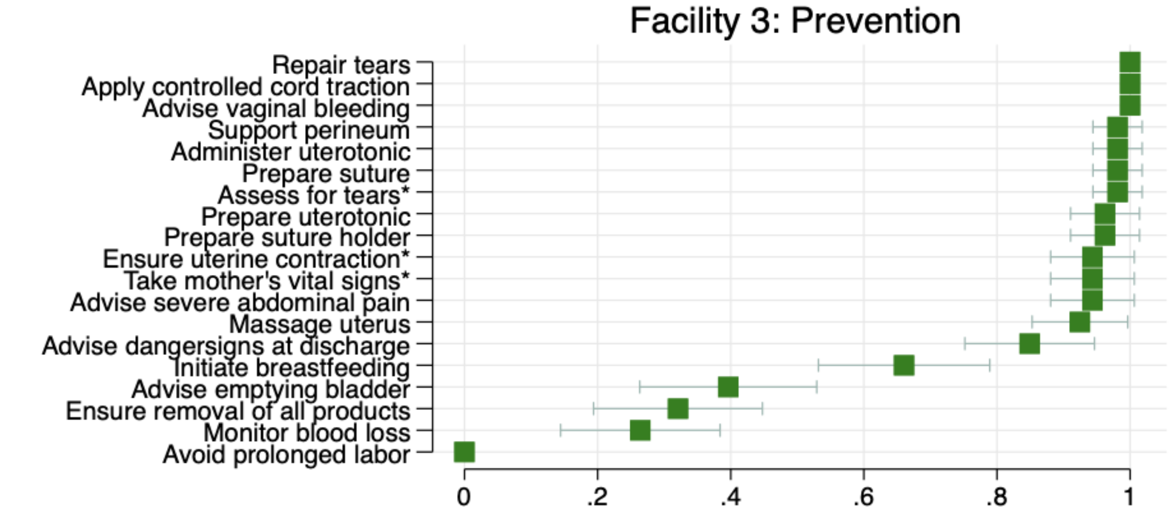

Management scores

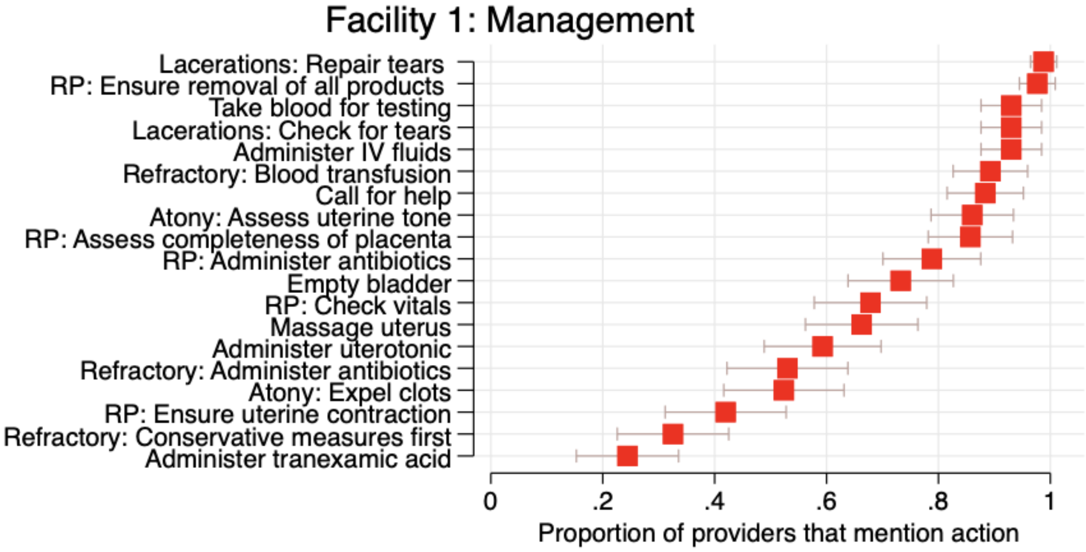

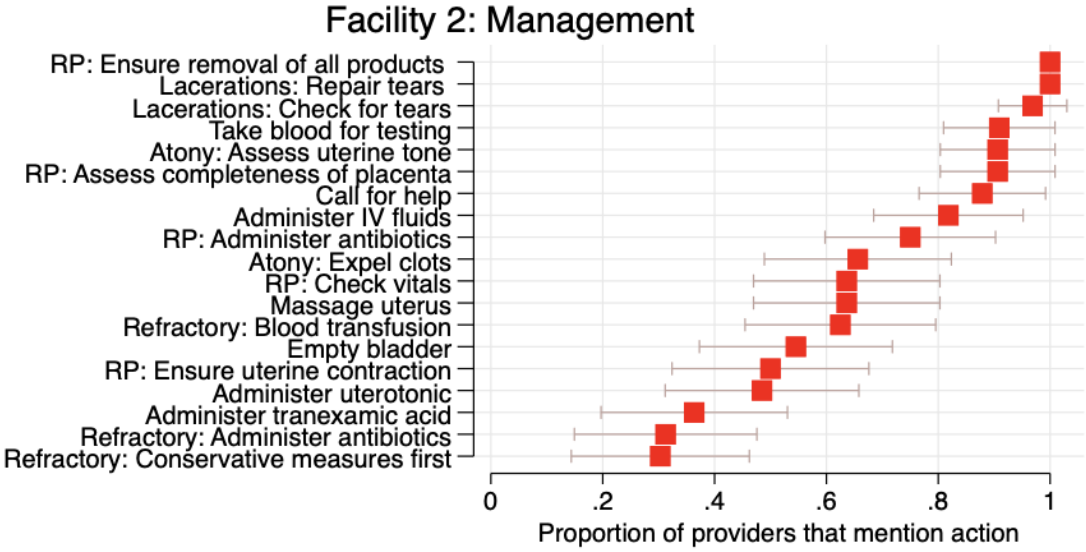

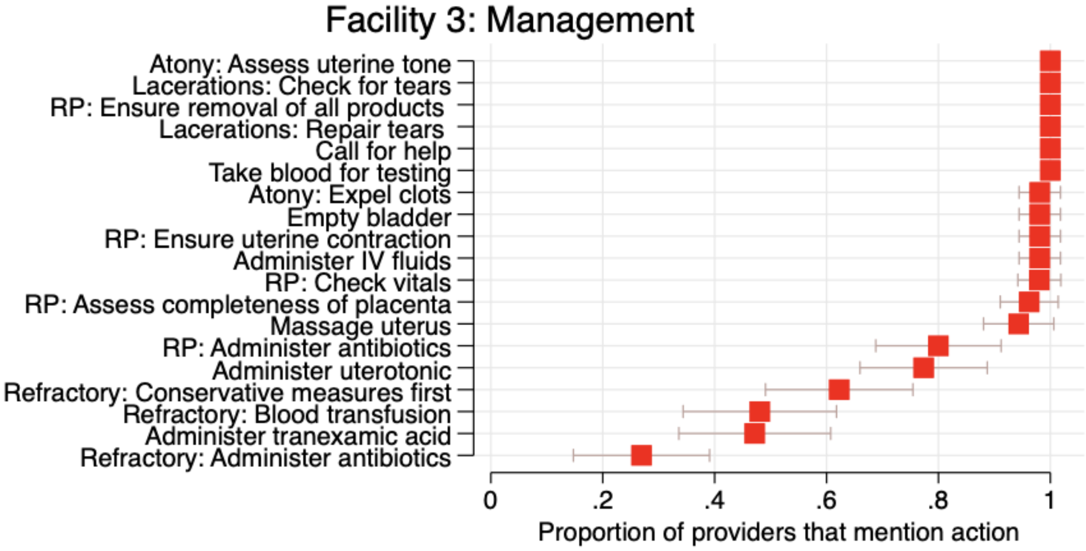

Supplement: Supplementary file 6 — Additional file 6. Facility-level comparisons of scores for each domain. [file 12884_2022_5128_MOESM6_ESM.pdf]
