# Supplementary material for: Health care providers’ knowledge of clinical protocols for postpartum hemorrhage care in Kenya: a cross-sectional study
Source: BMC Pregnancy Childbirth. 2022 Nov 10;22:828. doi: 10.1186/s12884-022-05128-6 (PMC9647972; doi:10.1186/s12884-022-05128-6)
Supplement: Supplementary file 7 — Additional file 7. Differences in scores by domain across desired improvements. [file 12884_2022_5128_MOESM7_ESM.pdf]

Additional File 7: Health care provider knowledge of clinical protocols for postpartum

Differences in scores by domain across desired improvements

		Assessment	Prevention	Management
		**More support from supervisors**		
Yes	N=121	0.81 (0.12)	0.73 (0.13)	0.77 (0.15)
No	N=43	0.81 (0.11)	0.67 (0.12)	0.69 (0.16)
p-value		*0.77*	*0.022*	*0.001*
		**Improvements in knowledge updates/training**		
Yes	N=163	0.81 (0.12)	0.71 (0.13)	0.76 (0.15)
No	N=7	0.84 (0.07)	0.67 (0.12)	0.65 (0.15)
p-value		*0.46*	*0.37*	*0.086*

Notes: This table shows the average scores of providers by domain across whether they would like to see specified improvements at their respective facilities or not. P-values are from the normal distribution for differences across categories based on the continuous score value.
